# Routine Orogastric Tube Placement Reveals an Unexpected Diagnosis Resulting in a Change in Therapy: A Case Report

**DOI:** 10.7759/cureus.104631

**Published:** 2026-03-03

**Authors:** Nitin Wadhwa, Arundati Iyer, Sabina Khan, Meera Gangadharan

**Affiliations:** 1 Anesthesiology, Critical Care and Pain Medicine/Pediatric Anesthesiology, Children's Memorial Hermann Hospital/UT Health, Houston, USA; 2 Biological Sciences, University of Southern California, Los Angeles, USA; 3 Anesthesiology, Critical Care and Pain Medicine, Children's Memorial Hermann Hospital/UT Health, Houston, USA

**Keywords:** inflammatory bowel disease, magnetic resonance enterography, orogastric tube misplacement, paraesophageal hernia, pediatric anesthesiology

## Abstract

We describe the case of a pediatric patient scheduled for a magnetic resonance enterography (MRE) requiring oral contrast, and in whom an orogastric tube (OGT) was placed after the induction of anesthesia. Unexpectedly, OGT placement required three attempts, although the final placement was smooth. This prompted the anesthesiologist to order a chest radiograph, which was suspicious for an esophageal perforation. Further investigation with an esophagram revealed a previously undiagnosed paraesophageal hernia and an intrathoracic stomach and ruled out an esophageal perforation. A reduced dose of contrast was administered to decrease the chances of aspiration because of the intrathoracic position of the gastro-esophageal junction, which results in an incompetent lower esophageal sphincter. The procedure was completed uneventfully. This case highlights the importance of confirming the OGT location before use. The case also underscores the need for anesthesiologists to perform a complete evaluation if OGT placement presents unusual challenges.

## Introduction

Orogastric tubes (OGTs) and nasogastric tubes (NGTs) are routinely placed by anesthesiologists, and placement is usually uneventful. There are a few cases of misplaced tubes described in the literature resulting in serious complications [1-3]. However, complications are rare, and most anesthesiologists will never have the experience of misplacing an OGT or NGT.

In pediatric anesthesia, the tubes are usually placed after the induction of general anesthesia, using a blind technique. In some circumstances, they may be placed before the induction of anesthesia, such as in pyloric stenosis, patients with esophageal dysmotility, full stomach, etc., when the risk of aspiration is high. Placement is usually confirmed by the aspiration of gastric contents when suction is applied to the tube.

We present the case of an OGT placement under anesthesia in a toddler, that was suspected to have caused an esophageal perforation, although further investigation with the administration of water-soluble contrast revealed a different diagnosis.

## Case presentation

The patient was a three-year-old girl with a history of significant microcytic anemia, previously hospitalized twice with hemoglobin levels of 2.1 g/dl and 7.2 g/dl, respectively. The presence of positive fecal occult blood prompted workup for GI bleeding and inflammatory bowel disease. This included an EGD, colonoscopy, and a Meckel's nuclear medicine scan, all of which failed to identify the cause of the anemia.

She was referred for MRE under general anesthesia as a final test to rule out inflammatory bowel disease (IBD). As the patient refused to drink the contrast, a 12F OGT was placed with the patient in the supine position using blind technique after induction of general anesthesia and endotracheal intubation. 

**Figure 1:**
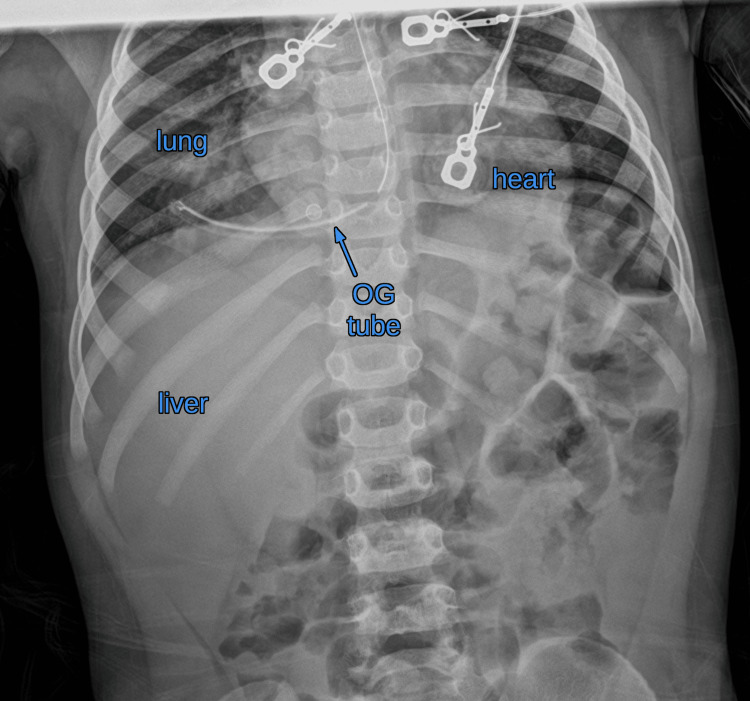
X-ray (anteroposterior view) of the lower chest and abdomen showing the orogastric (OG) tube turning towards the right side.

**Figure 2:**
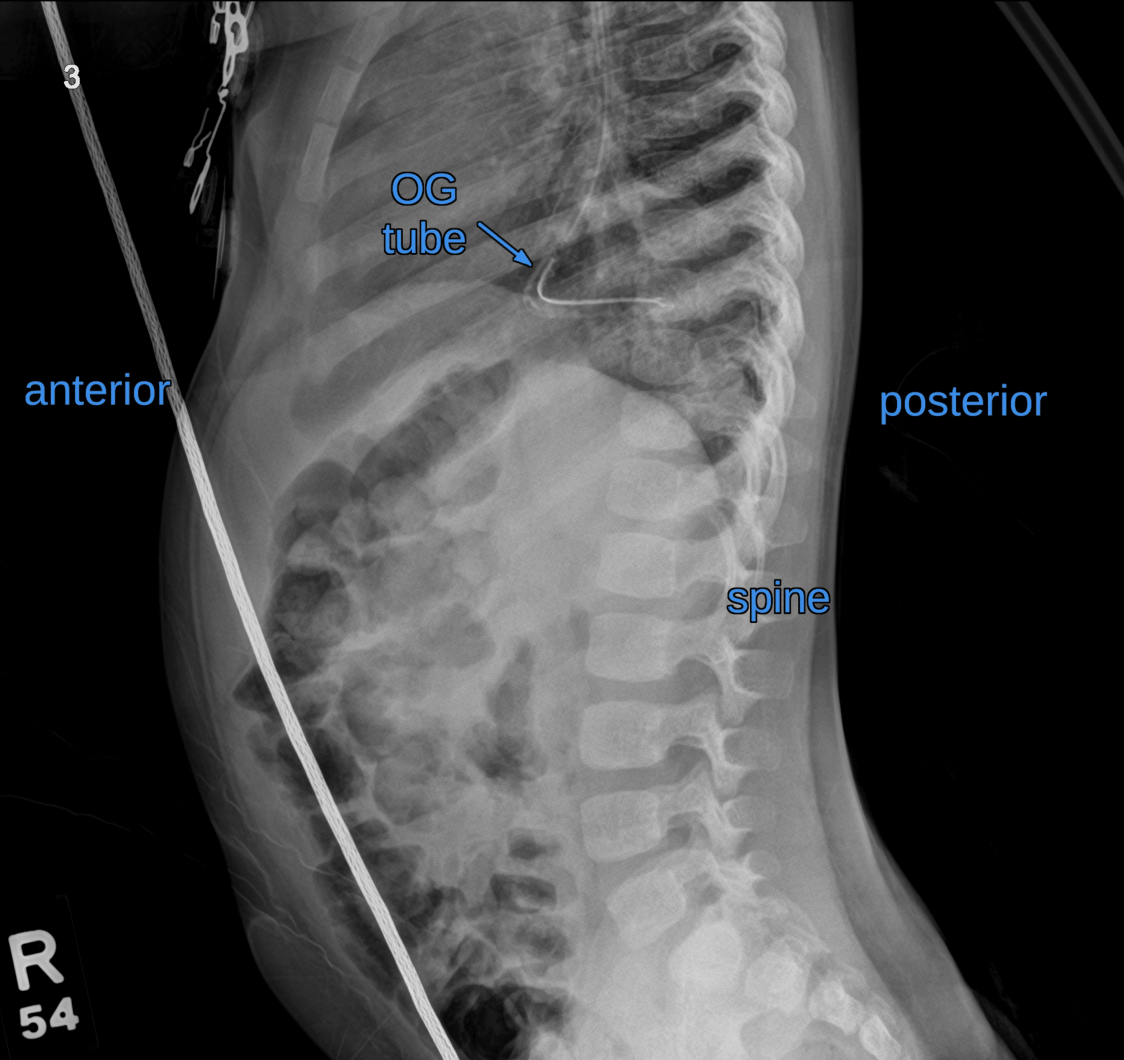
X-ray (lateral view) showing the orogastric (OG) tube with its tip towards the spinal column.

Initial OGT placement was met with resistance, and the tube was withdrawn. A second attempt was similarly unsuccessful. On the third attempt, the OGT passed smoothly without resistance. Given the unusual difficulty with placement, the anesthesiologist ordered a radiograph before administering contrast. The initial anteroposterior X-ray showed the OGT to be directed into the right hemithorax instead of following its typical course into the stomach (Figure 1). A subsequent lateral chest radiograph revealed posterior angulation of the tube directed from the distal esophagus toward the spine (Figure 2). These findings raised concern for a possible esophageal perforation with the tip being in the right lower lobe or in the right lower pleural space.

**Figure 3:**
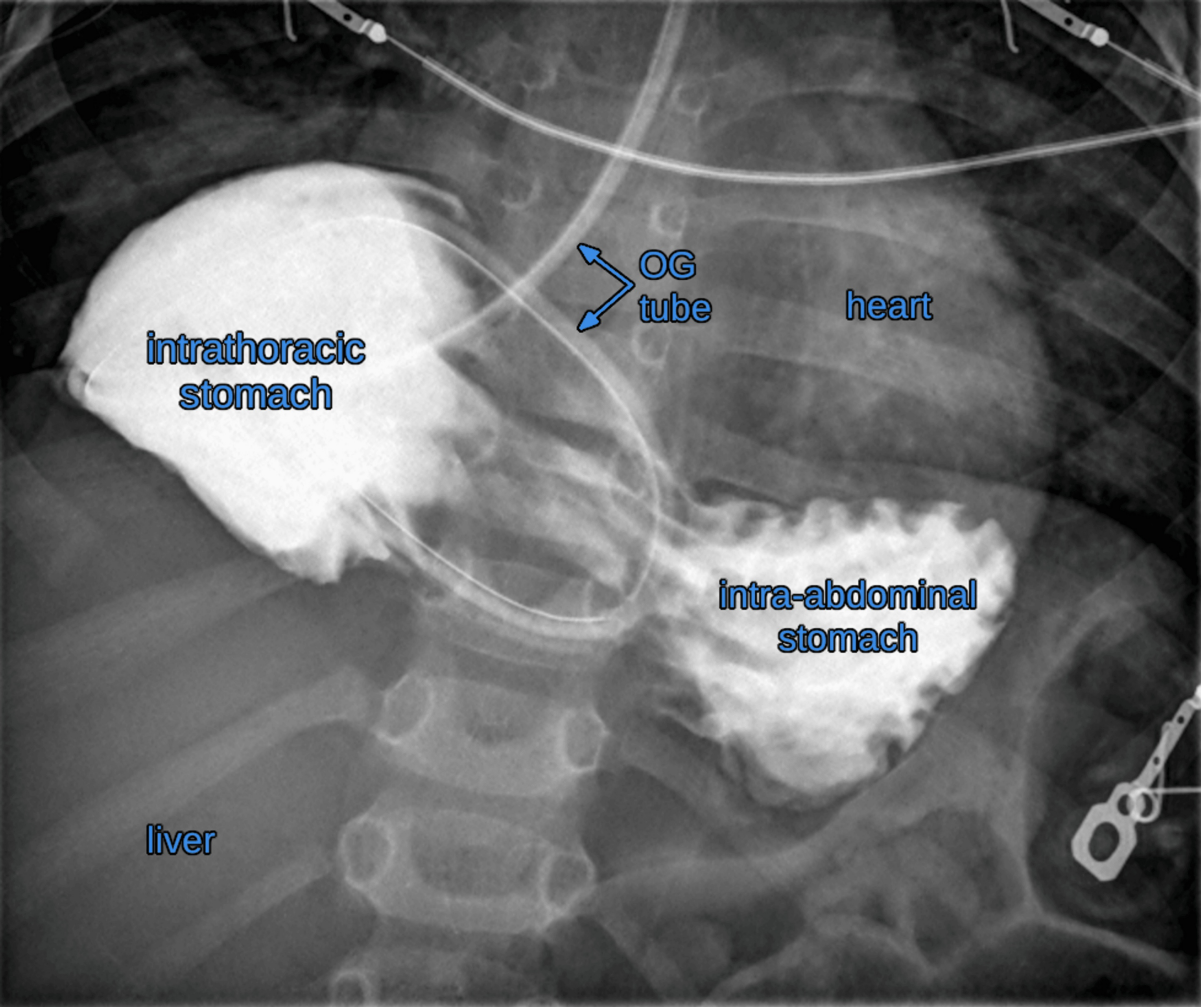
Esophagogram showing the stomach saddling the midline without any extravasation of contrast outside the stomach. OG: orogastric

This caused a lot of concern. Discussions were held with radiology, general surgery, a second pediatric anesthesiologist, and a cardiothoracic surgeon. The team elected to further evaluate the position of the OGT with an esophagram, using iodinated oral contrast. The esophagram ruled out any esophageal perforation, including intrapleural or mediastinal placement, but instead revealed a paraesophageal hernia with an intrathoracic stomach. The fundus of the stomach was herniated to the right side of the thorax; which was where the orogastric tube was seen (Figure 3). 

Following confirmation of the diagnosis, a reduced dose of oral contrast was administered. The usual dose of contrast for an MRE is 10 ml/kg. This child was given a smaller dose, although the exact dose is unknown. The MRE proceeded uneventfully, with the patient hemodynamically stable throughout the case. The child was extubated awake at the end of the procedure. Although the MRE did not specifically show IBD, it did show the paraesophageal hernia, which can cause chronic iron deficiency anemia from small, unseen bleeding erosions. A few weeks later, the patient underwent elective surgical repair of the hernia and had an uncomplicated perioperative course.

## Discussion

The prevalence of IBD in children in the United States doubled from 33 per 100,000 in 2007 to 77 per 100,000 in 2016 [4]. Approximately 10% of all cases of IBD begin during childhood. IBD is a chronic condition characterized by periods of exacerbation and remission. Current guidelines recommend that treatment targets include mucosal healing in addition to symptomatic improvement, for better long-term outcomes, including a reduced risk of colectomy.

Mucosal healing can be evaluated by endoscopy, intestinal ultrasound, computed tomography enterography, and MRE [5]. MRE is particularly advantageous as it is non-invasive and does not involve exposure to ionizing radiation. This feature is especially important in pediatric patients, who have a higher lifetime risk of radiation-induced malignancy compared to adults. Additionally, MRE aids in distinguishing between ulcerative colitis and Crohn’s disease and in assessing the extent and severity of disease in both conditions. Imaging guidelines for IBD in one tertiary care children’s hospital include MRE within four to six weeks of diagnosis, preferably before treatment has been initiated, and another MRE study at 12-18 months to evaluate the response to therapy. In cases of rapidly progressive illness, repeat MRE is suggested within three months of treatment initiation. MRE is also utilized to evaluate acute flare-ups when intestinal ultrasound findings are inconclusive [6].

MRE in young pediatric patients presents challenges, including motion artifacts, inability to drink large volumes of oral contrast, and difficulty with on-demand breath holds. As a result, many of these MREs are performed under general anesthesia, often necessitating OGT placement by the anesthesiologist for the administration of oral contrast. Although OGT placement is routine in anesthetic practice for surgical procedures and gastric decompression, the procedure is not without risk. Esophageal perforation following OGT or NGT placement has been reported in both adults and children, often following OGT and NGT placement, resulting in prolonged hospitalization and requiring interventions such as esophageal stenting and chest tube placements [1-3]. Traumatic insertion or malposition can result in complications, including epistaxis, sinus infections, pneumothorax, aspiration, intracranial or pulmonary placement, and esophageal or tracheobronchial perforations. Administering medications and/or feeds through a misplaced gastric tube may exacerbate these complications. These complications may be life-threatening and necessitate surgical intervention, particularly if there is a delay in diagnosis [1-3].

Anesthesiologists typically confirm OGT placement by smooth passage of the tube and aspiration of gastric contents. In cases of abdominal surgery, appropriate placement may also be confirmed intraoperatively by the surgical team. However, radiographic confirmation of OGT or NGT placement is not routinely performed by anesthesiologists. Alternative verification techniques, such as measuring pH or examining the color of aspirated contents, carbon dioxide detection, and auscultation over the abdomen with air insufflation, all have limitations and leave room for error. Chest X-ray remains the gold standard for verification of OGT/NGT placement. 

Paraesophageal hernias (PEH) are the rarest type of diaphragmatic hernia. They may be congenital or acquired. Acquired PEHs typically occur as a consequence of a gastroesophageal surgery or trauma. There are four types of congenital paraesophageal hernias. Type 1 is a sliding or hiatal hernia, in which the gastroesophageal junction (GEJ) is displaced cephalad above the diaphragm, while the gastric fundus is in the normal intra-abdominal location. In type 2 PEH, the gastric fundus herniates through the diaphragm, while the GEJ remains in the normal location. Type 3 PEH involves both the gastric fundus and the GEJ herniating into the thoracic cavity. Type 4 PEH is characterized by herniation of additional organs into the thoracic cavity, such as the spleen, colon, and stomach. Patients may be asymptomatic or may present with gastroesophageal reflux, anemia, vomiting, or recurrent respiratory infections. Gastric volvulus can be a dangerous complication of PEH [7]. In this case, the patient presented with anemia of unknown etiology and was found to have type 3 PEH. The intrathoracic location of the GEJ increases the risk of gastroesophageal reflux. Awareness of the patient’s anatomy resulted in a reduced dose of contrast and possibly prevented pulmonary aspiration of oral contrast.

Our case highlights several learning points for anesthesiologists. Pediatric MRE for children often requires placement of an OGT for the administration of contrast. Anesthesiologists may consider obtaining a chest radiograph to confirm the appropriate gastric tube location before administering contrast through it. Notably, in 2005, the National Health Service (NHS) in the United Kingdom issued a patient safety alert mandating the use of radiographs or pH indicator strips to confirm accurate placement of NGTs prior to administering feeds through them [8]. In subsequent statements, the NHS listed the administration of fluids into the respiratory tract or pleural cavity via a mispositioned gastric tube as a “never event”. A 2016 NHS document outlined steps that organizations should take to optimize patient safety regarding OGT and NGT placements. Interestingly, this report cites misinterpretation of radiographs by inadequately trained personnel as a common cause of error [8].

A consensus statement from the American Society for Parenteral and Enteral Nutrition makes several recommendations for NGT and OGT safety. These include education, appropriate methods of placement (depth of insertion should be length of the nose to ear to xiphoid, along the midline to the umbilicus), and indications for radiographic confirmation [9].

Complications related to misplaced gastric tubes under anesthesia are likely under-reported. In the United States, a misplaced NGT or OGT is a reportable event in only a few states [9]. This case and other published reports [4-6] underscore the need to standardize the process of verification of the location of OGTs and NGTs placed under anesthesia before administering substances through them.

## Conclusions

Location of OGTs must be verified before using them, including when they are placed under anesthesia for a procedure such as an MRE. If OGT placement is challenging, a complete evaluation must be done, including X-rays and contrast studies, if needed, to reach a diagnosis.
